# The prevalence of left ventricular thrombus among patients with low ejection fraction by trans-thoracic echocardiography

**DOI:** 10.12669/pjms.36.4.1972

**Published:** 2020

**Authors:** Nouradden Noman Aljaber, Zohoor Ali Mattash, Sultan Abdulwadoud Alshoabi, Fahad Hassan Alhazmi

**Affiliations:** 1 Nouradden Noman Aljaber, MD Faculty of Medicine, Sana’a University, Sana’a, Republic of Yemen; 2 Zohoor Ali Mattash, MD Military Cardiac Center, Sana’a, Republic of Yemen; 3 Sultan Abdulwadoud Alshoabi, MD, Department of Diagnostic Radiology Technology, College of Applied Medical Sciences, Taibah University, Kingdom of Saudi Arabia; 4 Fahad Hassan Alhazmi, PhD, Department of Diagnostic Radiology Technology, College of Applied Medical Sciences, Taibah University, Kingdom of Saudi Arabia

**Keywords:** Echocardiography, Ejection Fraction, Left Ventricular Thrombus, Spontaneous contrast

## Abstract

**Background and objectives:**

Ejection fraction (EF) is a measurement of heart function that reflects the portion of pumped out blood from the filled left ventricle per each heartbeat. The current study aimed to investigate the prevalence of left ventricular thrombus in patients with EF lower than 35% by using Transthoracic Echocardiography (TTE).

**Methods:**

In this prospective study, 82 cardiac patients underwent TTE procedure in order to assess the presence of left ventricular thrombus (LVT) from January 1^st^ to December 31st 2017 at the Military Cardiac Centre in Sana’a, Yemen.

**Results:**

Out of 82 patients enrolled in this study, the mean age was 49.13 ± 14.8 years and 87.8% were male. The mean of EF was 31.16% and LVT was found in 6.1%. The spontaneous contrast was seen in 25.6% of patients indicating strong relationship with low EF (p < 0.001). Among patients with low EF, ischemic heart disease (IHD) was identified in 50%, hypertension in 30.5%, diabetes mellitus (DM) type 2 in 23.2%, and hyperlipidemia 12.2%. Exactly 80% of LVT were detected in IHD patients with dilated cardiomyopathy (DCMP) and 80% of detected LVT were apical in site.

**Conclusion:**

Cardiac patients with low ejection fraction developed left ventricular thrombosis, and most of the affected patients were ischemic heart disease with dilated cardiomyopathy. Interestingly, spontaneous contrast was found high significantly in these patients, which may reflect the continuous process of thrombus formation.

## INTRODUCTION

Ejection fraction (EF) is the portion of pumped out oxygen-rich blood from a filled left ventricle to the aorta per each heartbeat. It measures the pumping ability and function of the heart. The cutoff of normal EF value is poorly-defined, however 50% of volume of blood in the left ventricle (LV) is considered as the lower boundary of normal.^1^ The American Society of Echocardiography (ASE) and the European Association of Cardiovascular Imaging (EACVI) consider the lowest limits of EF in male and female as 52% and 54% respectively.^2^ Left ventricular ejection fraction (LVEF) is a significant parameter for LV systolic function, which can be utilized as an independent predictor for mortality events after acute ischemic stroke.^3^ Based on the functional statues of the heart, heart failure (HF) is classified into: HF with preserved EF (HFpEF) and HF with reduced EF (HFrEF) that are occurred when EF is equal to or more than 50% and less than 40% respectively.^4^,^5^ LV thrombus is a serious complication of severe LV systolic dysfunction. The pathophysiology of the LV thrombus is related to endothelial injury, hypercoagulable state, and stasis of blood flow secondary to myocardial infarction (MI), chronic heart failure, and dilated cardiomyopathy (DCMP).^6^ LV apical thrombus is associate with low EF (35%) and also with apical aneurysm.^7^ The heart is a significant source of systemic emboli, and the most common cardiogenic source of emboli are atrial fibrillation (AF), LV thrombus, and prosthetic valves.^8^ LV apical thrombus is a serious complication of systolic dysfunction in patients with acute MI that is a risk of systemic embolization.^9^ EF is measured by assessing the changes in lumen of the LV.^10^ Transthoracic echocardiography (TTE), and/or trans-esophageal echocardiography (TEE) plays an essential role in evaluation, diagnosis and plan management of patients with low EF.^8^ TTE has 100% specificity for detecting LV thrombus.^11^

This study is intended firstly to assess the incidence of LV thrombus in patients with low EF, and secondary to assess the relationship between common habits in Yemeni population such as khat chewing, smoking, shamma using with reduced EF of the heart. Khat is a plant that is chewed for its stimulant action which is popular in Yemen.^12^

Shammah is a snuff of smokeless tobacco that is used in the middle east especially Saudi arabia, Sudan and Yemen.^13^ In this study, we used TTE as it is the most common imaging technique used to assess heart diseases. Based on our knowledge, this is the first study intended to assess the prevalence of LV thrombus in low EF patients.

## METHODS

### Patient selection

This cross-sectional, prospective study was conducted at Military Cardiac Center in Sana’a, Yemen. Data of 82 patients who underwent echocardiography in the outpatient clinic from January 1^st^ to December 31st 2017 and met the inclusion criteria of this study were collected. The inclusion criteria were:

1. Patients with EF less than 35% and more than 12 years old age.

The Exclusion criteria were:

1. Patients with rheumatic heart disease (RHD) who were under anticoagulant drugs.
2. Patients with atrial fibrillation (AF).

### Ethical approval

This study was approved by the Cardiac Center Research ethics committee. Full institutional ethical approval was obtained before beginning the study. Verbal consent from each patient was also obtained before examination. Confidentiality of all patient information was assured.

### Variables assessed

A pre-tested questionnaire was prepared to include all relevant information. Researcher through direct interview filled the questionnaire with the patients. Explanation of the study purpose was explained to each patient, and consent was obtained. The following variables are included: sociodemographic, clinical history for cardiac disorders causes, risk factors such as hypertension (HTN), diabetes mellitus (DM), smoking, hyperlipidemia, drug in-use and disease duration, severity and complications.

Physical examination was performed for each patient looking for signs of congestive heart failure (CHF) as increase jugular venous pressure (JVP), hepatomegaly, lower limb edema. Investigations were done included complete blood count (CBC), cardiac enzymes, liver function tests (LFT), renal function test (RFT), electrocardiogram (ECG), and chest x-ray (CXR). Patients with EF less than 35% underwent another TTE to assess the presence of left ventricular thrombus (LVT).

### Echocardiography (ECHO) procedure

The ECHO procedure was performed with the same ultrasound imaging machine (Philips I E 33, Australia) using a standard protocol. All selected patients were examined by the same cardiologist with 25 years’ experience in echocardiography.

LVT was defined as a hyperechoic mass with definite margins adjacent to the myocardium on multiple plane views through the cardiac cycle. Regional wall motion abnormalities were considered present whether hypokinesia, akinesia or dyskinesia were observed in at least two segments of the left ventricular wall. Left ventricular function was assessed by echocardiographic examination. LV end diastolic and end-systolic volumes and ejection fraction were determined from apical 2 and 4 chamber views using Simpson`s biplane formula according to recommendation of the American Society of echocardiography: EF=(EDV-ESV)/EDV.^2^

**EF:** Ejection fraction

**EDV:** End Diastolic Volume (ml)

**ESV:** End Systolic Volume (ml)

### Statistical analysis

Data analysis was performed using SPSS version 21 (IBM). Data are presented as frequency and percentage for continuous variables and mean ± Standard Deviation (SD) for descriptive variables. Quantitative data were analysed using One sample student t-test, and qualitative data were compared using Chi square test. P-value was assumed to be significant when P < 0.05.

## RESULTS

In total, 82 patients who had EF <35% included in this study. The mean age at diagnosis was 49.13 ± 14.8 years; 87.8% were male and 12.2% were female. Echocardiographic findings showed the mean of EF was 31.16% and LVT was present in 6.1%, which is statistically not significant (p = 0.61). The spontaneous contrast picture was seen in 25.6% of patients (p < 0.001) -(Table-I). Among patients with low EF, IHD was identified in 50%, hypertension in 30.5%, DM type 2 in 23.2%, and hyperlipidemia 12.2% (Table-II).

**Table-I:** Shows findings of TTE in patients with <35% EF.

Patients with EF less than 35%	Frequency	%
EF < 30	21	25.6
EF 30-35	61	74.4

*Patients with positive findings:*	*Frequency*	*%*

LVT	5	6.1
Spontaneous contrast	21	25.6

EF: Ejection fraction, LVT: Left ventricular thrombosis.

**Table-II:** Shows the comorbidities of the patients with <35% EF.

Comorbidities	Frequency	%	p-value
Ischemic heart disease (IHD)	41	50	0.07
Hypertension (HTN)	25	30.5	0.21
DM type-2	19	23.2	0.65
Hyperlipedemia	10	12.2	0.98

Khat chewer and smokers were the most common popular habits in the patients of low EF then Khat chewing only but no significant relationship (p=0.96) (Table-III). Exactly 80% of LVT were apical, 80% were in ischemic heart disease patients with dilated cardiomyopathy (Table-IV).

**Table-III:** Shows the common bad habits of the patients.

Habits	Frequency	%
Smokers	2	2.4
Khat chewers	23	28
Khat chewers + Smokers	29	35.4
Khat chewers + Smokers + Shammah users	10	12.2
Khat chewers + Smokers + Alcoholic	1	1.2
Khat chewers + Shammah users	3	3.7
None	14	17

Total	82	100

**Table-IV:** Details of thrombus among the 5 patients of LVT in the patients of < 35% EF.

No.	Site	Nature	Size (mm)	EF	Disease	Co-morbidities
1	Apical	Organized	N/A	29	DCMP	IHD + ↑ lipid
2	Apical	Organized	N/A	29	DCMP	IHD
3	Apical	Organized	7x12	34	DCMP	IHD + DM
4	Apical	Organized	12x8	32	DCMP	IHD + DM+ HHD
5	Mural	Organized	14x40	25	HHD	HHD

DCMP: Dilated cardiomyopathy, HHD: Hypertensive heart disease, IHD: Ischemic heart disease, DM: Diabetes mellitus.

**Fig.1:**
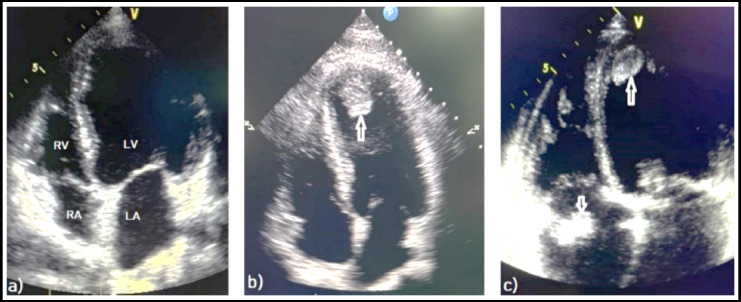
Transthoracic echocardiography images shows a) normal all cardiac chambers b) left ventricular thrombus (arrow), and c) left ventricular thrombus (long arrow) and right atrial thrombus (short arrow). **LV,** left ventricle; **RV,** right ventricle; **LA,** left atrium; **RA,** right atrium.

## DISCUSSION

LV thrombus is a serious complication of heart failure. In this study, we assessed the prevalence of LV thrombus in patients with heart failure with low EF. Disertori et al., reported that patients with LVEF less than 35% have a high risk of death due to evolution of HF.^14^ In this study, we found LV thrombus in 6.1% of patients and that may be a source of serious emboli to any other critical organ including the brain. This result is consistent with the results of Watanabe et al., who reported significant relationship between cerebral micro bleeds and low left ventricular EF.^15^ In this study, 50% of patients with low EF was associate with IHD. This result is consistent with Vedin et al., who reported the high prevalence of IHD in patients with HFrEF with increased risk of new IHD events.^16^ The result are also consistent with Rahmayani et al., who reported that low left ventricular EF affects the clinical outcomes of IHD patients.^17^ In this study, we found that 80% of LV thrombus were apical. This result is consistent with Adar et al., who reported that LV thrombus often formed in the LV apex.^18^ The result also consistent with Kaolawanich et al., who reported that apical area index is a new index in patients with LV systolic dysfunction to predict LV thrombus.^19^ The apical site of thrombi was explained by Benito who reported that the apex of the ventricle is the most prone to blood stasis.^20^ In this study, 80% of the patients with low EF were khat chewers and 80% of LV thrombus were in IHD. This result is consistent with Al-Motarreb et al., who reported that khat chewing is a risk factor for acute MI by causing coronary spasm. Mega et al., reported that heavy khat chewers are at higher risk factor of acute MI than moderate khat chewers.^21^,^22^

### Limitations of the study

It was a single-center study and the sample size was limited by 82 patients.

## CONCLUSION

Cardiac patients with low ejection fraction developed left ventricular thrombosis and most of the affected patients were of dilated cardiomyopathy. Interestingly, spontaneous contrast was significantly high in patients with low ejection fraction which is a continuous process of thrombus formation. This make starting anticoagulant therapy in this group of cardiac patients is an individual decision based on patient and physician discussion.

### Author`s contribution

**NMA:** Conceptualization, and data collection.

**ZAM:** data analysis.

**SAA:** writing the original draft and is responsible for integrity of research.

**FHA:** manuscript review & editing.

**Abbreviations**
EF: ejection fraction,
ASE: American society of echocardiography,
EACVI: European association of cardiovascular imaging,
LV: left ventricle,
LVEF: left ventricular ejection fraction,
HFpEF: heart failure with preserved ejection fraction,
HFrEF: heart failure with reduced ejection fraction,
MI: myocardial infarction,
DCMP: dilated cardiomyopathy,
AF: atrial fibrillation,
TTE: Transthoracic echocardiography,
TEE: trans-esophageal echocardiography,
RHD: rheumatic heart disease,
HTN: hypertension,
DM: diabetes mellitus,
CHF: congestive heart failure,
JVP: jugular venous pressure,
CBC: complete blood count,
LFT: liver function tests,
RFT: renal function test,
LVT: left ventricular thrombus,
ECG: electrocardiography,
LVT: left ventricular thrombus,
EDV: end diastolic volume,
ESV: end systolic volume,
SPSS: statistical package for the social sciences,
IBM: international business machines,
SD: standard deviation.
